# The role of selected egzo- and endogenic agents on preinitiation process of bladder cancer

**DOI:** 10.1186/1897-4287-13-S2-A14

**Published:** 2015-11-26

**Authors:** A Iwaniuk, M Owczarek, M Pietrusiński, E Borkowska, M Rozniecki, M Borowiec, M Sosnowski, J Kocki, B Kałużewski

**Affiliations:** 1 GENOS Non-Public Healthcare Institution, which is a member of the Polish Technology Platform for Innovative Medicine, of the National Centre for Research and Development, Poland; 2 Chair of Clinical and Laboratory Genetics Department of Clinical Genetics of Medical University of Lodz Genetic Outpatient Clinic, Lodz, Poland; 3 I Urology Clinic of Military Medical Academy University Teaching Hospital - Central Veterans' Hospital, Lodz, Poland; 4 Department of Clinical Genetics of Medical University of Lublin, Poland; 5 Non-Public Outpatient Clinic of Urology - "Lekarze Urolodzy Marek Rożniecki i Partnerzy", Lask, Poland

## 

The pilot study included 25 patients with symptoms of recurrent infection of the urinary tract.

The results of urine sediment cytology (presence of atypical cells = cytology +), which was performed in this group (positive in 92%), were accepted as an additional criterion for inclusion to the study group. The control group was consisted of the 78 female patients, from whom we obtained DNA from cervical smears, while realizing one of the local Screening Programs and these patients reported no complaints from the genitourinary system. In the DNA samples obtained from patients of the study group, the infection of the high-risk Human Papilloma Virus (HPV=HPV+) was confirmed in 65%, the same group of patients were tested on the presence of high-risk HPV in material derived by catheterization of the urinary bladder, the result was positive (HPV +) in 32% cases. In the control group the presence of high-risk HPV DNA in samples derived from cervical smears were found in 20.6% of patients. In these group of HPV (+) patients, the DNA samples obtained from urine sediment, the methylation profile of five genes: p16INK4a, p14ARF, DAPK, RASSF1A, APC was analysed. It was based on an assessment of the presence or absence of specific "U" or "M" bands, which are the visualization of the PCR products after the electrophoresis in a polyacrylamide gel followed by staining using ethidium bromide. Two patients (DNA samples from the urine sediment: cytology + ; HPV +) demonstrated the presence of abnormal methylation profile. In the first case the result concern the p16INK4a gene, the second - the APC gene. In one case, during diagnostic transurethral bladder cystoscopy, verified by histopathological examination, the presence of squamous cell metaplasia was revealed. The second case was diagnosed with chronic cystitis. It appears that urine cytology supplemented by the DNA test for the presence of HPV and a molecular test for the presence of methylation of selected genes may provide a valuable indication for bladder cystoscopy. The study was financed by Genos NON-Public Healthcare funds.

